# Nutritional composition, sensory acceptability and shelf-life evaluation of wheat–corn chaff composite cake

**DOI:** 10.3389/fnut.2026.1808630

**Published:** 2026-04-30

**Authors:** Joe Dare Nyefene, Patricia Glago

**Affiliations:** Department of Hotel, Catering and Institutional Management, Dr. Hilla Limann Technical University, Wa, Ghana

**Keywords:** composite flour, consumer preference, maize by-products, sensory acceptability, shelf-life modeling

## Abstract

**Background:**

In many low- and middle-income countries (LMICs), like Ghana, cereal-based baked products such as cakes are widely consumed due to their affordability and convenience. However, conventional wheat-based cakes are often characterized by low micronutrient density, limiting their contribution to adequate nutrition.

**Objective:**

This study aimed to develop wheat–corn chaff composite cakes and evaluate their nutritional composition, sensory acceptability, and shelf-life stability.

**Methods:**

Composite cakes were produced by substituting wheat flour with 0, 10, 15, and 20% corn chaff. Proximate composition was determined using standard AOAC methods, while mineral content (iron, zinc, and magnesium) was analyzed using Atomic Absorption Spectrophotometry. Sensory evaluation was conducted among 75 panelists using a hedonic scale and Relative Importance Index. Shelf-life stability was predicted using moisture-based accelerated models.

**Results:**

Incorporation of corn chaff significantly increased ash content, indicating improved mineral density, while maintaining desirable carbohydrate and protein levels. Fiber content also improved significantly from 0.58 ± 0.07 to 2.15 ± 0.12. The composite cakes provided appreciable amounts of essential minerals (Iron = 2.87 ± 0.06 mg/100 g, Zinc = 2.70 ± 0.14 mg/100 g, Magnesium = 95.0 ± 1.41 mg/100 g), demonstrating improved micronutrient composition compared to conventional wheat cake. Sensory evaluation showed significant differences among formulations (*p* < 0.05), with the 10% corn chaff cake receiving the highest overall acceptability (4.933 ± 0.251). Shelf-life was slightly reduced (8 months) compared to wheat flour (10 months), likely due to higher moisture retention.

**Conclusion:**

Incorporation of 10% corn chaff significantly improves the micronutrient quality of wheat-based cakes while maintaining consumer acceptability and appreciable shelf-life stability. This approach offers a practical strategy for enhancing the nutritional value of commonly consumed baked products and promoting sustainable utilization of maize by-products.

## Introduction

Cereal-based baked products constitute significant part of human diets which enjoy large patronage in Low-and Middle–Income countries (LMICs) due to common availability, affordability and acceptability by customers. Cakes are among these products that are untilized by people of all ages due to their convenient and appealing sensory qualities. However, the conventional wheat-based cakes are primarily high in energy and offer only modest amounts of dietary fiber and vital micronutrients, making them ineffective in addressing the micronutrient deficiencies that are common in many LMICs. It is reported that cakes prepared from refined wheat flour are “limiting in protein, minerals and dietary fiber, iron, zinc and calcium compared to whole wheat flour alternatives (1, 2). For example, fiber content in fortified bakery products was reported to vary from 1.3–12.1 g/100 g in enriched cookies, cassava composite flour biscuits: 2.77–3.44 g/100 g, and 11.80 g/100 g in whole meal rye bread. Also, composite biscuits with pearl millet + orange peel showed improvement in crude protein from 11.70–13.41%, crude fiber from 11.44–16.24% and ash content: 4.50–5.59% (3). A workable food-based approach to increasing nutrient consumption of populations is to diversify the ingredients in such commonly consumed food items to improve their nutritional content (4).

Micronutrient deficiencies, especially those affecting iron, zinc, and magnesium remain a significant public health concern. Iron deficiency continues to be the most prevalent dietary issue worldwide, particularly in the majority of West African developing countries and it affects cognitive development, occupational capacity and immune function (5). Zinc deficiency affects development and immunological function and magnesium is required for numerous metabolic and enzymatic processes, including energy metabolism and neuromuscular function (6). Food-based therapies that include micronutrient-rich substances into commonly consumed foods are increasingly recognized as effective alternatives to pharmaceutical fortification and supplementation (7). The use of composite flour technology has shown to be a successful strategy for enhancing the nutritional value of wheat-based foods while lowering reliance on imported wheat. This technology involves the partial substitution of wheat flour with locally available plant materials or agro-industrial by-products, thereby enhancing nutritional composition and promoting sustainable resource utilization (8). Using the by-products from the processing of maize for value addition offers a promising avenue for food product innovation in sub-Saharan Africa, where maize is extensively grown and wheat production is limited.

The results of a previous study reported the levels of ash, iron, zinc and magnesium in maize bran and associated by-products, indicating the concentration of minerals in their outer kernel layers (9). Consequently, there may be nutritional benefits in incorporating maize by-products into human food products, particularly in terms of raising the consumption of dietary fiber and mineral content. The moisture retention and storage properties of baked foods may also be impacted by its high water-holding capacity (10).

Maize chaff, a by-product when maize is milled has a proximate composition that makes it a useful ingredient for product innovation and food fortification. Along with moderate amounts of residual protein and low fat, it is usually characterized by relatively high dietary fiber and ash content, indicating a strong source of indigestible carbohydrates and vital minerals (11). This is in contrast to refined wheat flour, which frequently has low levels of minerals and fiber because of processing losses. Therefore, adding maize chaff to cake recipes has the potential to improve the nutritional profile by boosting fiber content, enhancing density of some minerals and promoting improved digestive health (12). In addition, its utilization supports waste valorization and sustainable food production by converting an underused agricultural by-product into a functional food ingredient (13). Therefore, combining maize chaff with traditional cake recipes offers a viable way to create baked goods that are more nutritious while encouraging creative use of locally accessible resources.

However, adding fiber-rich ingredients to baked goods may change their physicochemical and sensory characteristics, such as texture, flavor, appearance and general appeal (54). Unlike bread or biscuits, cakes depend largely on a delicate balance of fat, moisture and structure for consumer acceptance. As a result, determining suitable substitution levels that improve nutritional quality without sacrificing sensory qualities is thus critical (14). Therefore, customer preference ranking and sensory assessment are essential components in the development of composite food products.

Another vital consideration to make in food product development is the shelf life of a product, as it affects its quality, safety and marketability. Moisture content and water activity have a major impact on the microbial stability and physicochemical deterioration of cakes during storage (15). The shelf life and microbiological stability of baked goods like cakes are significantly impacted by moisture content and water activity (aw). Water activity indicates the availability of free water for microbiological growth and chemical reactions, whereas moisture content describes the overall amount of water in the product (16). While high water activity (aw > 0.85) encourages the growth of spoilage microorganisms, such as bacteria, yeasts, and molds, products with high moisture content but low water activity may nonetheless have longer shelf lives (16).

Cakes are prone to microbiological deterioration, especially mold growth during storage, due to their generally intermediate to high moisture content and water activity (17). Reduction in water activity through formulation adjustments (e.g., sugar and fat content) or packaging can significantly enhance shelf stability by limiting microbial proliferation (18). Furthermore, texture might be impacted by moisture migration within the cake matrix during storage, eventually resulting in staling or dryness (19).

Another significant factor influencing shelf life is storage temperature. Due to rapid microbiological growth and physicochemical changes, cakes kept at room temperature (about 25–30 °C), which is typical in tropical areas like Ghana, typically have a shorter shelf life (20). Refrigeration (around 4–10 °C) can extend shelf life by slowing microbial activity and delaying spoilage, although it may also accelerate staling due to starch retrogradation (21). Freezing (< −18 °C) is most effective for long-term preservation cakes, as it significantly inhibits microbial growth and maintains product quality when properly packaged (22),

The addition of maize chaff, which has a high capacity to hold moisture, may have a positive or negative effect on shelf-life behavior, depending on the formulation amount. Nevertheless, nothing is known about the shelf-life modeling of composite cakes made with maize by-products in the literature (23), thus making it an area to research into. Even though a few studies have looked at the use of alternative flours and cereal brans in baked products, these mainly focused on bread and biscuits, paying little attention to cakes. Additionally, recent research frequently assesses sensory qualities or nutritional composition of composite food products separately, ignoring customer choice and shelf-life evaluation (24). The combined effects of adding corn chaff on the nutritional value, sensory acceptability, customer preference and shelf-life stability of wheat-based cakes are therefore not well documented empirically.

Concerns about sensory quality and storage stability limit the use of maize chaff in human food items, despite the fact that it contains significant levels of minerals like iron, zinc and magnesium. When substitution levels are not appropriately optimized, high fiber inclusion has been linked to unfavorable changes in texture, flavor and appearance. Moreover, limited information exists on how corn chaff affects moisture dynamics and shelf-life behavior in cake products, which are particularly sensitive to changes in formulation. Also, food processors’ capacity to identify ideal formulation levels that strike a balance between nutritional benefit and product acceptability and stability is hindered by this disjointed approach. Consequently, a thorough analysis of wheat-corn chaff composite cake that incorporates consumer choice, sensory acceptability, nutritional composition and shelf-life assessment is a necessity. This work closes these gaps by producing wheat-corn chaff composite cakes at various substitution levels and carefully evaluating their proximate composition, mineral content, sensory attributes, consumer preference ranking and shelf-life aspects. Closing this gap will encourage the sustainable use of agro-processing by-products, improve the nutritional value of frequently consumed foods and offer evidence-based recommendations for the use of maize chaff as a functional ingredient in bakery products. The study seeks to explore the following objectives:

1. To develop wheat–corn chaff composite cake and evaluate its nutritional composition, sensory acceptability, and shelf-life stability.
2. To determine the effects of varying levels of corn chaff incorporation on the proximate composition, mineral content, consumer preference, and shelf life of wheat-based cake.

The findings provide scientific evidence that maize chaff can be added to baked goods while preserving the stability and appeal of the finished product.

## Materials and methods

### Wheat flour-corn chaff composite preparation

Wheat flour was purchased from the local market and was sifted to remove lumps and foreign particles, improve aeration and to ensure uniform particle size. Corn chaff was also collected from the local corn mill operators, further dried, sieved and blended in electric blender to convert it into almost powder state to obtain fine particle size. The composite was then prepared by carefully blending predetermined grams of the wheat flour with corn chaff to get a uniform mixture.

### Cake production

#### Ingredients and formulations

Three different samples of cakes were produced in addition to wheat flour alone cake as the standard or control. The ingredients used included wheat flour, corn chaff, margarine, sugar, egg, Nutmeg, baking powder, vanilla essence, pineapple essence and milk. Apart from corn chaff which had varying grams in the various cake sample, the quantities of all other ingredients remained same in all samples. The formulation used was 100 g of wheat flour mixing with 10 g, 15, 20 g and 0.00 g of corn chaff, respectively, to obtain a total of four different cake samples.

#### Production procedure

The wheat flour was sieved with a 40 mesh (~420 μm) particle size net and the 100 g measured into mixing bowl. About 4 g of baking powder and 4.5 g of grated nutmeg were added to the flour and caused to mix well. 100 g of margarine was rubbed into the mixture to obtain a sandy texture. A total of 50 g of sugar was added and mixed thoroughly into the flour mixture using a fork. Also, 1 egg, 40 mL of milk and about 5 g of vanilla and 5 g of pineapple essence were then added to the mixture and stirred with a fork to have a uniform mixture. The fork was used to scoop the mixture into cake tins and baked in a gas-operated oven at a temperature of about 180–200 °C for 20 min. The same ingredients and procedure was used to produce the wheat flour alone and corn chaff alone cakes for comparisons for proximate composition and mineral content analysis.

### Proximate analysis

The Association of Official Analytical Chemists, or (53) recommendations were followed for all proximate analyses of maize chaff cake, wheat flour cake and their composite mixture cake. Protein, carbohydrates, moisture, ash, crude fibre and fat content were the parameters analyzed. Carbohydrates were calculated by difference as described by (25). Values for all parameters were determined on triplicate bases and the averages reported and compared with standard values for reliability as follows:

Protein determination: Protein content was determined using the Kjeldahl method according to Association of Official Analytical Chemists (53). Approximately 2 g of the maize cake sample was weighed into a Kjeldahl digestion flask and digested with concentrated sulfuric acid (H₂SO₄) in the presence of a catalyst mixture (potassium sulfate and copper sulfate) to accelerate the digestion process. The mixture was heated at 350–420 °C until a clear solution was obtained. After digestion, the solution was cooled and diluted with distilled water. The digest was neutralized with sodium hydroxide (NaOH), and the liberated ammonia was distilled into a boric acid solution containing mixed indicator. The distillate was then titrated with standardized hydrochloric acid (HCl) to determine the nitrogen content. The crude protein content was calculated by multiplying the total nitrogen by a conversion factor of 6.25.

Calculations:


$$\%N=\frac{(V_{a}-V_{b})\times N_{A}\times 1.401}{W}$$

%Protein=%N×6.25.

Nitrogen Free Extract (NFE):


%NFE=100−(%Moisture+%Fat+%Crude Fiber+%Protein+%Ash)

%NFE=100−(%Moisture+%Fat+%CrudeFiber+%Protein+%Ash)

Moisture Content and Total Solids: The Oven-drying process was adopted at 105 °C for 5 h until a steady weight was recorded (26, 27).

Calculation:


$$\%\text{Moisture}=\frac{\text{Weight loss}}{\text{Initial weight}}\times 100$$

%Total Solid=100−%Mixture

Ash Content: The ash concentrations were of the samples determined after the samples were incinerated in a muffle furnace for 2 h at 600 °C (28).


$$\%\mathrm{Ash}=\frac{\mathrm{Ash}\;\text{weight}}{\text{Sample weight}}\times 100$$

Fat Content (Soxhlet Extraction): After refluxing roughly 5 g of dried samples with petroleum ether at 40–60 °C for 6 h, the percentage of fat was calculated using the method outlined by (29) and referenced in (29) as follows:

Calculation:


$$\%\mathrm{Fat}=\frac{\mathrm{Fat}\;\text{weight}}{\text{Sample weight}}\times 100$$

Crude Fiber Determination: As stated by (55) and referenced in (30), the fiber content was established by sequentially digesting samples with 1.25% H₂SO₄ and 1.25% NaOH, then filtering, drying and ashing the samples. Percentage crude fiber was then calculated by:

Calculation:


$$\%\text{Crude Fibre}=\frac{\text{Residue loss}}{\text{Sample weight}}\times 100$$

Carbohydrates: The carbohydrate level was estimated by method of difference as:


%Carbohydrates=100−(%Moisture+%Fat+%Protein+%Ash)

%Carbohydrates=100−(%Moisture+%Fat+%Protein+%Ash)

### Mineral content analysis

The mineral composition of the sample cakes was evaluated using spectrophotometry (AAS) through the Atomic Absorption method of wet digesting. Each cake sample, weighing approximately 0.25 g, was digested using 1 mL of perchloric acid, 2.5 mL of concentrated HNO₃, and 2.5 mL of concentrated HSO₄. Following heating, the digested sample was cooled, diluted to 50 mL with deionized water and then filtered. After that, the mixture was inhaled into a flame, where the metal ions absorbed light at element-specific wavelengths. The quantity of each mineral (iron, zinc, and magnesium) in the sample is directly correlated with the amount of light absorbed. Mineral concentrations were quantified by comparing the absorbance values with those of standard calibration solutions and results were expressed as mg per 100 g or mg per kg of the sample cake (31).

### Sensory evaluation and acceptability test

Three samples of cakes were prepared and compared with control (without corn chaff) (32). The formulation ratio for the four samples were 100:00, 100:10, 100:15 and 100:20 with the 100 g representing wheat flour and the 0.00 g, 10 g, 15 g and 20 g representing corn chaff, respectively (33).

Students of Dr. Hilla Liman Technical University who were randomly selected were trained on how to evaluate the samples and were then made to taste the various samples and rank them using a questionnaire to describe the appearance, aroma, taste, texture/feel and general acceptability. The samples were randomly presented to the panelists with a randomization order generated using Compusense Cloud software. The sample size used for the evaluation was 75 (34)

### Shelf-life analysis

The shelf-life of wheat flour and wheat–corn chaff composite flour was evaluated using an accelerated shelf-life testing (ASLT) approach based on moisture-driven deterioration kinetics. Samples were packaged in airtight polyethylene bags and stored under controlled environmental conditions at elevated temperatures (35, 45, and 55 °C) and a constant relative humidity of approximately 75% to accelerate deterioration (35). Moisture content was selected as the primary deterioration indicator due to its strong influence on flour stability, caking and susceptibility to microbial growth.

Quality parameters monitored during storage included moisture content, texture, color, and flavor. Moisture content and texture were measured weekly, while color and flavor were assessed biweekly. Microbial load (total viable count, yeast, and mold) was evaluated every 4 weeks. Shelf-life endpoint for flour samples was defined as the point at which moisture content exceeded acceptable limits or when significant deterioration in sensory and physical quality was observed.

The Arrhenius kinetic model was applied to describe the temperature dependence of the deterioration rate and to predict shelf-life under ambient conditions. To improve model fit and ensure linearity, a square-root transformation of the dependent variable (√Y) was applied. Additionally, a logarithmic transformation of moisture content (lnX) was used for the composite flour to better capture moisture-dependent deterioration kinetics, as such transformations are commonly applied in food shelf-life modeling to improve predictive accuracy when deterioration kinetics are moisture-dependent (36). Shelf-life estimates were obtained by extrapolating accelerated storage data to typical ambient storage conditions (25–30 °C).

For cake samples, shelf-life was determined through real-time storage studies. Freshly baked cakes were cooled to room temperature (25–30 °C), packaged in polyethylene bags and stored under ambient conditions (relative humidity ~70–80%). Analyses were conducted on the first day of storage day 0 and subsequently at 24-h intervals. Deterioration indicators included sensory attributes (appearance, aroma, taste, texture and overall acceptability) and microbiological quality.

Microbial analysis was performed using standard plate count methods to determine total viable count and yeast and mold levels. The microbiological acceptability limit was set at 1.0 × 10^3^ cfu/g for yeast and mold. Sensory evaluation was conducted by a semi-trained panel using a 5-point hedonic scale. The shelf-life endpoint for the cake was defined as the last day on which the product remained free from visible mold growth and maintained acceptable sensory scores (37).

### Statistical analysis

Descriptive statistics was used to evaluate differences between the standard wheat flour cake and the composite cake as well as the sensory preferences. All experiments were conducted in triplicate and the results of the statistical analysis were recorded as mean ± standard deviation (SD). To compare mean values of carbohydrate, protein, fat, ash, moisture and minerals (iron, zinc, magnesium) among the samples (wheat flour cake, corn chaff cake and wheat flour-corn chaff composite cake), as well as the hedonic scale ratings (appearance, aroma, taste, texture, acceptability) with HSD to compare means across sample ratios (100:00, 100:10, 100:15, 100:20), one-way Analysis of Variance (ANOVA) was conducted. Tukey’s HSD (post-hoc test) was applied to identify significant pairwise differences between groups, with significance set at *p* < 0.05. Differences in means are denoted using superscript letters (a, b, c), where distinct letters within a column indicate statistically significant differences. Non-linear regression models (M1: square root-Y logarithmic-X; M2: square root-Y) were used to predict shelf life (months) based on moisture content. For participants’ most preferred cake sample, the Relative Importance Index (RII) analysis was conducted (Table 1).

**Table 1:** Formulation formula for composite mixture of wheat–corn chaff cake.

Samples (g)	Sample 4	Sample 3	Sample 2	Sample 1
Ratios	100:00	100:20	100:15	100:10

## Results

Mineral composition analysis was conducted for the wheat flour cake, corn chaff cake and the wheat–corn chaff composite cake. The results shows that wheat cake contained significantly higher magnesium (117.0 ± 1.41) and zinc (2.51 ± 0.13) than corn chaff cake (*p* < 0.05). The Corn chaff cake also demonstrated lower concentrations of iron, zinc and magnesium contents compared with wheat flour cake. The composite sample cake however showed intermediate mineral values, with iron (2.87 ± 0.06) and magnesium (95.0 ± 1.41) falling between those of wheat flour and corn chaff cakes. Zinc content in the composite remained comparable to wheat flour, indicating retention of zinc levels despite corn chaff incorporation (Table 2).

**Table 2:** Mineral composition of wheat flour, corn chaff and wheat–corn chaff cake.

Sample	Iron	Zinc	Magnesium
Wheat flour cake	3.25 ± 0.21^b^	2.51 ± 0.13^b^	117.0 ± 1.41^c^
Corn chaff cake	2.71 ± 0.16^a^	1.72 ± 0.15^a^	81.0 ± 2.83^a^
Composite cake	2.87 ± 0.06^a^	2.70 ± 0.14^b^	95.0 ± 1.41^b^

Atomic Absorption Spectrophotometry (AAS) was used; values are means ± standard deviations of duplicate determinations; means with different superscripts in the same column differ significantly (*p* < 0.05).

With regards to the proximate characteristics of the various cake samples, wheat flour cake had the highest carbohydrate (69.92 ± 0.25) and protein (10.70 ± 0.33) contents, while corn chaff cake exhibited significantly lower carbohydrate and protein levels but higher ash content (4.01 ± 0.08). The wheat–corn chaff composite cake maintained a high carbohydrate content (69.22 ± 0.50) with moderate protein (8.72 ± 0.29), reflecting the contribution of both ingredients. Ash and moisture contents of the composite cake were significantly higher than those of wheat flour cake, indicating increased mineral content and water retention capacity following corn chaff incorporation. The composite cake also demonstrated significant improvement in fiber content compared with the conventional wheat flour cake (Table 3).

**Table 3:** Proximate composition of wheat flour, corn chaff and their composite (% dwb).

Sample	Carbohydrate	Protein fiber	Fat	Ash	Moisture
Wheat flour cake	69.92 ± 0.25^a^	10.70 ± 0.33^a^ 0.58 ± 0.07^c^	9.67 ± 0.05^a^	1.24 ± 0.34^c^	8.46 ± 0.35^c^
Corn chaff cake	10.17 ± 0.51^c^	4.76 ± 0.15^c^ 3.45 ± 0.15^a^	1.65 ± 0.04^b^	4.01 ± 0.08^a^	9.78 ± 0.17^b^
Composite cake	69.22 ± 0.50^b^	8.72 ± 0.29^b^ 2.15 ± 0.12^b^	1.86 ± 0.22^b^	3.07 ± 0.11^b^	11.02 ± 0.36^a^

Explanation: A standard AOAC approach was used, composite = Wheat flour + Corn chaff. Values are mean± standard deviation on triplicate determinations. Means with different superscripts in the same column differ significantly (*p* < 0.05).

To determine the amount of corn chaff addition which will be most accepted by the market, sensory evaluation of the samples produced with varying corn chaff substitution levels in a composite cake was conducted. Significant differences (*p* < 0.05) were observed among samples for all sensory attributes. The 100 g:10 g formulation recorded the highest scores for appearance, aroma/flavor, taste, texture and overall acceptability. In contrast, the 100 g:20 g sample had the lowest sensory scores across most attributes, while the 100 g:15 g sample demonstrated moderate acceptability. The control sample (100 g:00 g) generally received lower scores than the 100 g:10 g and 100 g: 15 g formulations, indicating improved sensory performance at lower levels of corn chaff incorporation. The details are presented in Table 4.

**Table 4:** Sensory characteristics and acceptability of wheat–corn chaff composite cake.

Attribute					
Sample	Appearance	Aroma/flavor	Taste	Texture/feel	General acceptability
100:00	2.467 ± 0.502^b^	2.267 ± 0.741^b^	2.680 ± 0.756^b^	2.227 ± 0.815^c^	2.520 ± 0.578^b^
100:10	4.533 ± 0.502^a^	4.493 ± 0.503^a^	4.893 ± 0.311^a^	4.693 ± 0.464^a^	4.933 ± 0.251^a^
100:15	2.453 ± 0.527^b^	2.587 ± 0.496^b^	2.613 ± 0.676^b^	2.947 ± 0.543^b^	2.667 ± 0.600^b^
100:20	1.933 ± 0.704^c^	1.853 ± 0.711^c^	1.320 ± 0.573^c^	2.133 ± 0.759^c^	2.001 ± 0.735^c^

A 5-point hedonic scale was used to conduct the sensory evaluation with sample ratios of 100:00, 100:10, 100:15 and 100:20 representing the proportions of corn chuff to wheat flour in a composite cake. Values are presented as mean ± standard deviation. Means with different superscripts in the same column differ significantly (*p* < 0.05).

To further confirm the acceptability of the wheat flour-corn chaff composite cake, Relative Importance Index (RII) test was run using the scores for each sample cake. The 100:10 sample ranked first with the highest RII value (78.563%), followed by the 100: 15 and third position was control sample (100:00). The 100:10 sample ranked third, while the 100:20 sample was least preferred. These results indicate variations in consumer preference across substitution levels, with moderate corn chaff inclusion showing higher overall preference (Table 5).

**Table 5:** Ranking of participants’ most preferred sample.

Sample	Formulation (g)	RII (%)	Rank
Sample 1	100:10	78.56	1
Sample 2	100:15	63.25	2
Sample 4	100:00	2.42	3
Sample 3	100:20	6.34	4

RII, Relative Importance Index. Formulations of samples are expressed in grams.

The wheat flour and wheat–corn chaff composite flour were subjected to shelf-life evaluation based on moisture-dependent predictive models using the Accelerated Shelf-Life Testing (ASLT). The wheat–corn chaff composite flour exhibited a shorter shelf life of approximately 8 months compared with 10 months for wheat flour alone. The reduced shelf life of the composite flour was associated with its higher moisture content, as reflected in the logarithmic moisture-based model. On the other hand, the shelf life of the cakes using the storage study combined with and sensory analyses revealed that the wheat flour cake showed a shelf stability of 6 days under room temperature storage and 9 days under refrigeration while that of the composite cake remains stable for 4 days at room temperature and 7 days under refrigeration. These results suggest that corn chaff incorporation influences moisture dynamics and storage stability of the composite flour (Table 6).

**Table 6:** Predictive models for shelf-life estimation of wheat flour cake and wheat flour–corn chaff composite cake.

Sample	Predictive model	Model type	R^2^/goodness of fit	Storage temp.(°C)	Estimated shelf life
Wheat Flour–Corn Chaff Composite Cake	Y = (2.82131–2.85042lnX)^2^	Square-root Y – Logarithmic X Model	0.94	25–30 (ambient)	8 months
Wheat Flour Cake	Y = (2.69926–0.930382X)2	Square-root Y Model	0.92	25–30 (ambient)	10 months

Y = Predicted shelf life (months), X = Moisture content of cake samples (% wet basis), M1 = Square root–Y logarithmic–X model and M2 = Square root–Y linear–X model.

## Discussion

Incorporation of corn chaff into wheat-based cake formulations in this study demonstrates a practical approach to valorizing cereal by-products while maintaining the nutritional quality, sensory properties and shelf-life stability of the cake. The results of the study are in line with previous studies which reported the effects of composite flours and fiber-enriched maize chaff inclusion on mineral composition, proximate characteristics, sensory acceptability and storage behavior of baked products. It has been reported that partial wheat flour substitution with corn-derived powders demonstrated only limited effects on cake characteristics, with sensory quality generally remaining unaffected (11). Additionally, a study found that adding up to 20% maize fiber to cakes had a notably positive effect on their sensory attributes (38). It was shown that adding sweet corn residue to cakes might raise their contents of carotenoids, dietary fiber, folate and vitamin E levels while keeping their sensory attributes comparable to control cakes (12). Roughages and fiber are essential components of human nutrition because they facilitate digestion and help avoid constipation. These consistent results from various experiments support corn chaff’s potential as a useful cake ingredient. Therefore, the improved fiber content of the composite cake upon the addition of corn chaff not only significantly altering the nutritional quality, sensory characteristics and shelf-life stability, it offers an avenue for corn chaff, which is often wasted to be utilized in a manner that contributes to human nutrition and income generation for domestic users of corn products.

The noticeably high magnesium and zinc concentrations observed in wheat flour cake as opposed to corn chaff cake are consistent with earlier findings showing that refined wheat flour often maintains appreciable amounts of these elements, even after losses during the process of milling. For example, it was discovered that the aleurone layer of wheat grains had higher concentrations of phloem mobile elements such as Mg, K, P, Fe, Zn and Cu (39). Additionally, it was noted that whole wheat flour products have higher mineral content than refined wheat flour and that grain anatomy affects the distribution of minerals in grains, with outer fibrous layers (such as corn chaff) having fewer bioavailable minerals and more dietary fiber (39). Iron, zinc and magnesium concentrations were lower in maize chaff cake, as the maize is mainly made up of the fibrous outer layers of maize kernels. This is consistent with studies which reported that cereal husks and chaff are richer in dietary fiber than in bioavailable minerals.

Rather than showing a nutritional deficit, the wheat–corn chaff composite cake showed intermediate mineral levels, reflecting a minimal diluting effect. Zinc content of the composite cake was statistically equivalent to that of wheat flour cake, indicating that a moderate amount of maize chaff inclusion does not substantially lower zinc levels. Thus, the results are consistent with past research showing that incorporating corn chaff into wheat flour-based baked products preserves important micronutrient levels and may even provide functional benefits (40). Comparable patterns have been noted in composite flours made from maize or wheat bran by-products, where functional qualities were enhanced but important micronutrient levels were preserved through partial substitution (41). In rural communities in low- and middle-income countries like Ghana where many households are food insecure, this finding is profound given the widespread zinc and iron deficiencies (42). Because corn chaff is readily available and almost cost nothing to procure, its usage in this manner will help poor households meet their iron and zinc needs. Also, from a public health perspective, this finding is particularly relevant as zinc and iron deficiencies remain widespread in low- and middle-income countries (42).

The proximate composition analysis indicate that corn chaff cake had a much higher amount of ash, whereas wheat flour cake was characterized by higher protein and carbohydrate contents. This pattern is in line with other research that found higher ash levels in cereal by-products. According to a study, the composite flour product had a slight loss in protein but retained high amounts of carbohydrates due to a subtle compositional interaction. This pattern is consistent with what is known about the composition of grain by-products, particularly the composition of outer layers that are rich in minerals and contribute to higher ash values (43). The proportionate contribution of both ingredients was reflected in the wheat–corn chaff composite cake, which maintained high carbohydrate levels with a minor protein loss making it an ideal food product for resource limited families struggling to meet energy and protein needs.

Increased moisture content indicates better water-holding ability, which is typically associated with fiber-rich ingredients. The composite also display a noticeably high ash level while maintaining a relatively moderate mineral profile. This finding aligns with earlier research that found that composite flours boosted with cereal by-products had higher mineral content and water-holding capacity, but that there were complicated trade-offs in the performance of baked products (44). The findings of another study revealed that composite flours exhibited noticeably higher fiber, ash and moisture levels than control samples (45). While improved water retention may have a variety of uses in the baking industry by improving baked products’ crumb softness and freshness, it can also have a negative effect on product attributes like shelf-life stability (44). From an academic perspective, the findings support the body of knowledge regarding the functional effects of adding cereal by-products to refined flour matrices.

The findings of the sensory evaluation showed that all assessed attributes were strongly impacted by the level of corn chaff incorporation. The 100:10 formulation’s superior performance in terms of appearance, aroma, taste, texture and overall acceptability is in line with research that indicates that while excessive incorporation of fiber-rich cereal by-products results in unfavorable textural and flavor changes, low-to-moderate incorporation improves sensory quality (56).

While a 30% incorporation of corncob powder into other cereal products has been confirmed to have achieved high overall acceptability in instant cereal beverages, a similar study found that bran additions up to 20% maintained adequate sensory quality in the composite product (46). The results of this study are thus consistent with evidence in literature that suggests that a moderate inclusion of fiber (10–30%) can enhance sensory qualities such as flavor, texture and appearance, but that higher concentrations may result in a decline in product quality. This trend is consistent with a variety of corn-based products such as breads, snacks and beverages, suggesting a fiber incorporation principle that can be generalized.

Higher fiber content interfering with the formation of gluten networks may account for the decrease in acceptability shown at higher corn chaff substitution levels (100:15 and 100:20), as this interaction often results in a thicker crumb structure and decreased palatability (54). Critical substitution limits for many products have been validated by numerous other researches. For example, corn fiber becomes undesirable up to 20% incorporation level (56), pea/bean pod flour at 15% (47) and oat fiber beyond 20% (54). These findings underscore the importance of carefully optimizing by-product inclusion into baked products in order to strike a balance between nutritional benefit and sensory quality.

Relative Importance Index test conducted indicated that the 100:10 formulation was the most preferred sample, further validating the sensory findings. This suggests that consumers preferred and accepted the optimally fortified product over the control sample. Consumer tests of composite bakery items have shown similar results, with moderate integration levels leading to higher preference scores because of the perceived health benefits and better texture. It was discovered that biscuits fortified with moringa and green tea were generally well-received by panelists compared with the control samples (48). Another study revealed that rice cakes made with balanced fortification levels of fiber achieved optimal consumer satisfaction (49). This formulated corn chaff-added cake will thus provide an option for consumers of cake products with preference for more fiber inclusivity.

A similar study also found that composite cakes containing up to 15% fortifiers retained high sensory acceptability, suggesting that moderate substitution levels can improve nutritional profile without sacrificing sensory qualities (52). This suggests that low levels of maize chaff can be used for industrial purposes without compromising marketability, supporting the growing trend toward sustainable product development through the utilization of agro-industrial residues in value-added food products.

A shorter predicted shelf life for both the wheat–corn chaff flour and its composite cake was recorded compared to wheat flour alone and its cake, which is largely attributable to the higher moisture content in the composites. Moisture remains one major and critical determinant of microbial growth and physicochemical deterioration in flour-based products (50). The vulnerability of the composite flour and its cake to moisture-induced degradation was accurately reflected by the logarithmic moisture-based model employed in this work, thus providing a caution for the usage of such blends commercially. This was further corroborated by the shortened shelf stability observed with the composite cake (4 days) as against the wheat flour alone cake (6 days) under normal temperature storage conditions. Besides, comparable decreases in shelf life have been documented for baked goods and fiber-enriched flours, highlighting the necessity of better packaging, moisture management, or the use of natural preservatives (51). While this may be seen as a limitation for long-term storage, the industrial applications of the composite, particularly for short-to-medium shelf-life bakery purposes is still viable.

### Strengths, limitations and future research directions

The study provides a comprehensive review of wheat-corn chaff composite cake by evaluating nutritional composition, sensory quality, consumer preference and shelf-life behavior. The application of statistical validation and standardized analytical techniques improves the reliability of the results but the study is however limited by the absence of real-time storage experiments, mineral bioavailability evaluation and dietary fiber profile. Future research should use *in vitro* digestive models to examine fiber and mineral bioavailability, which are critical nutritional characteristics of cereal by-products. Additionally, processing methods such as fermentation or enzymatic treatment should be investigated as they may improve sensory quality at higher substitution levels. Also, long-term storage research using different packing materials could be utilized to improve shelf-life stability.

## Conclusion

This study demonstrates that corn chaff can be effectively utilized as a functional ingredient in wheat-based cake, providing a sustainable use of cereal by-products. Moderate incorporation enhanced fiber content while maintaining acceptable mineral composition and preserving key nutrients, including carbohydrates and protein. The composite cake also showed increased ash and moisture content, reflecting the contribution of corn chaff.

The 100:10 formulation achieved the best balance of sensory quality and acceptability, highlighting the importance of optimizing substitution levels. While the composite flour exhibited good storage stability (8 months), the composite cake had a shorter shelf life (4 days at room temperature), though still suitable for short- to medium-term use.

Overall, the findings highlight the potential of corn chaff to improve product functionality, sustainability, and nutritional value without compromising consumer acceptance, making it a viable option for product development and industrial application.

## Data Availability

The raw data supporting the conclusions of this article will be made available by the authors, without undue reservation.
